# Systematic review of parenting interventions in European countries aiming to reduce social inequalities in children’s health and development

**DOI:** 10.1186/1471-2458-14-1040

**Published:** 2014-10-06

**Authors:** Joana Morrison, Hynek Pikhart, Milagros Ruiz, Peter Goldblatt

**Affiliations:** Department of Epidemiology and Public Health, University College London, London, UK; Institute of Health Equity, Department of Epidemiology and Public Health, University College London, London, UK

**Keywords:** Review, Early intervention, Parenting, Inequalities, Health, Child development

## Abstract

**Background:**

Early child development influences many aspects of wellbeing, health, competence in literacy and numeracy, criminality, and social and economic participation throughout the life course. Children from disadvantaged groups have less possibilities of achieving full development. By providing a positive start for all children across the social gradient, improved developmental outcomes will be seen during later childhood and throughout their lives. The objective of this systematic review was to identify interventions during early childhood in countries from the World Health Organisation European Region in 1999–2013 which reduced inequalities in children’s health and development.

**Methods:**

A systematic review was carried out adhering to the PRISMA guidelines. The review examined universal, targeted and proportionate universalism interventions, programs and services using an electronic search strategy in PubMed and the International Bibliography of the Social Sciences [IBSS] databases. A further search was performed in the grey literature. Interventions were included only if they were aimed at children or their parents and had been evaluated.

**Results:**

We identified 23 interventions in total: 6 in the PubMed data base, 5 in IBSS and 12 in grey literature. All but 1 intervention-delivered in Sweden-were carried out in the United Kingdom and the Republic of Ireland. These aimed to improve parenting abilities, however, some had additional components such as: day-care provision, improving housing conditions and speech or psychological therapies. Programmes offering intensive support, information and home visits using a psycho-educational approach and aimed at developing parent’s and children’s skills showed more favourable outcomes. These were parenting behaviours, overall children’s health and higher level of fine motor skills and cognitive functioning. Child injuries and abuse were also reduced. Two interventions were universally proportionate and all others were aimed at a specific target population.

**Conclusions:**

Interventions with better outcomes and a higher level of evidence combined workshops and educational programmes for both parents and children beginning during early pregnancy and included home visits by specialised staff. Further evaluation and publication of early years interventions should be carried out also within a wider range of countries than just the UK and Ireland.

## Background

During child growth, neuron connections produce cognitive, motor, emotional, behavioural and social developmental skills [1]. Childhood risks associated with poverty or similar adverse conditions, such as lack of stimulation or excessive stress, affect brain development. The risks begin prenatally by influencing the foetal brain through exogenous factors that produce maternal stress [2]. Early child development [ECD] will influence many aspects of wellbeing, health, competence in literacy and numeracy, criminality and social and economic participation throughout the life course [3–5].

The early acquisition of skills is part of a developmental continuum and commences well before formal schooling [6]. By the time the child enters school, development will have already been influenced by family, neighbourhood and the broader societal level [7]. Family socioeconomic status [SES] is associated with a multitude of development outcomes [8], for example, it has been described that children of mothers with mental health problems were more likely to have negative behavioural, emotional, and peer outcomes [9]. Low family SES also produces obesity in childhood and adolescence and may exert a strong influence on socioeconomic status [10]. Children from disadvantaged groups are less likely to achieve a good level of development and have worse health outcomes [11]. Neighbourhood deprivation and the physical context also influence early child development [12, 13] and children from family backgrounds that pose multiple threats to their development tend to do better growing up in mixed socioeconomic neighbourhoods [14].

These different qualities of experience create social gradients in human developmental trajectories across the life course [15–17]. As described elsewhere, children from the 1970 British birth cohort survey assessed by tests of intellectual, emotional and personal development who were in the bottom SES quartile at 22 months were still there at age 10. High-SES children showed considerably more upward mobility and were more likely to be in the top quartile by age 10, even if they were in the bottom quartile at 22 months [18].

Evidence from intervention studies suggest that performance in the different domains of Early Child Development [ECD]-described as the development of physical, socio-emotional and language-cognitive capacities in the early years-[19] can be modified in ways which improve health, well-being, and competence in the long-term [8]. By providing a positive start across the social gradient, children will benefit from improved developmental outcomes during later childhood and throughout their life course as significant improvements in all domains of child development will influence later school achievement [20]. The overarching conceptual framework for this study: “Action across the life course”-as described in the Strategic Review of Health Inequalities in England post-2010-[21] shows that disadvantage starts before birth and accumulates throughout life. Therefore, action to reduce health inequalities must start during gestation and be carried out through the life of the child and into adulthood. This may be made effective by providing evidence-based interventions and delivery systems [20, 21].

Studies which have shown the importance of parenting activities across income groups and the social gradient [15] fostered through ECD programmes are not limited to cognitive gains, but also include physical, social, and emotional gains, all of which are determinants of health over the life course [3]. The quality of parent–child relationships is significantly associated with many outcomes relating to child health and development, including learning and social skills, mental health and health behaviours and remain influential into adulthood for social and behavioural outcomes. Parenting programmes offer valuable opportunities to positively influence child health and well-being through health-promoting environments, establishing good health behaviours, providing support for families and creating resilience [22]. Examples of early years programmes delivered in the USA have been well documented: “The Perry Preschool Project” delivered during 1962–1967 and the “High/Scope Preschool Curriculum Study” (1967–1970) which showed positive outcomes for test scores, high school completion, lower arrests and criminality, teenage pregnancies and higher home ownerships. The “Carolina Abecedarian Project” (1972–1985) and the “Syracuse Family Development Research Program” (1969–1975) had an impact on improving development and IQ scores [19, 23].

The economic return to these programs is high, especially when considering alternative policies that target children from disadvantaged environments or the policies targeting the young adults who emerge from them [24]. There is sufficient scientific rationale for early intervention [25], as social inequalities develop before birth it is more effective to deliver interventions not only in the early stages of the child’s life [26], but also before birth and has been established as a priority at the UN Convention on the Rights of the Child [7].

It has been argued as a principal recommendation in a previous study, that future reviews should focus on identifying interventions [17] and to our knowledge, there are few scientific reviews of interventions to tackle health and developmental inequalities in early child development focusing only on European studies. The majority include intervention studies carried out in the US, Canada and Australia or in low income and high burden countries [27, 28]. The WHO European Region includes countries with close to the best health and narrowest health gaps in comparison to other continents and has benefited from a sustained period of social cohesion, developed welfare states and high-quality education and health services [29]. However, inequalities still remain and are increasing in some countries, therefore the different set of conditions across Europe offers the possibility of evaluating evidence on the effectiveness of early interventions on families’ socio-economic conditions as well as the physical functioning and development of children in the early stages of their lives.

By reviewing this evidence, the objective of this systematic review was to identify relevant existing literature on interventions carried out during early childhood in countries within the Region during the years 1999–2013 which address children’s health and development. The review forms part of the DRIVERS Project (2012–2014) – a three-year research project funded by the European Union 7th Framework Programme focusing on three of the key drivers to reduce health inequities: early childhood development, employment and working conditions and welfare, income and social protection. It assesses the impact of policies and programmes to develop new methods and evidence and provide policy recommendations and advocacy guidance to reduce health inequalities within Europe. The research builds on the recommendations of the Commission on Social Determinants of Health (CSDH) [11], The Strategic Review of Health Inequalities in England post-2010 [21] and The Review of Social Determinants and the Health Divide in the World Health Organisation European Region [29] and seeks to contribute to the EU 2020 initiatives [30].

## Methods

### Data sources

The review examined the literature on early childhood interventions which have been defined elsewhere as experiences from conception to the start of statutory school [31]. The study followed the Centre for Reviews and Dissemination guidance for undertaking reviews [32] adhering to the PRISMA guidelines [33]. We applied the PICOCS (Population, Interventions, Comparisons, Outcomes, Context, Study Design) guidelines [34], to develop the search terms.

We carried out a search in PubMed’s database and the International Bibliography of the Social Sciences (IBSS) database to include research from the medical and social sciences. Table 1 shows the search strategies used in both databases. A further search was made in the following online grey literature databases and search engines: the National Institute for Health and Care Excellence’s (NICE) evidence database, the System for Information on Grey Literature in Europe (SIGLE) and Google. We used the keywords: “early years interventions to reduce inequalities in child health and development”. The inclusion criteria were: studies could be from any country in the World Health Organisation European Region; the interventions, programmes or services had to have undergone a formal evaluation, describing the methods used to evaluate the programme or intervention. Study designs included were: Randomised Controlled Trials (RCT), experimental and quasi-experimental studies, before and after evaluations and qualitative research assessments. Interventions had to show outcomes in child health and developmental domains and/or parenting as international evidence has supported that programmes and services at this stage of life are aimed at parents as well as children [31]. We included papers published in peer reviewed journals and reports in grey literature, published between January 1999-the publication year of the earliest article identified-and December 2013 and no articles were excluded due to language criteria. We excluded studies aimed at children over eight years of age; the latest compulsory age for schooling in the WHO European Region [35], as early years and childhood disadvantage is linked to disadvantage in adulthood, and poor adult health [4]. Therefore the review focused on interventions delivered during the early years, however some programmes had follow-ups during later stages. Systematic reviews which included interventions delivered outside WHO European Region were also excluded.

**Table 1:** **Search strategies for PubMed and IBSS**

Search Strategy PubMed	[[[[[[[[[[[[[[[[[[[[[[[[Policy[Title/Abstract]] OR intervention[Title/Abstract] OR parenting[Title/Abstract] OR community health planning[Title/Abstract] OR charities[Title/Abstract] OR child day care centres[Title/Abstract] OR foster home care[Title/Abstract] OR food assistance[Title/Abstract] OR government financing[Title/Abstract] OR tax exemption[Title/Abstract] OR family support[Title/Abstract] OR child health centres[Title/Abstract] AND [[[[[Child[Title/Abstract]] OR infant[Title/Abstract]] OR newborn[Title/Abstract]] OR early childhood[Title/Abstract]] OR prenatal[Title/Abstract]] AND [[[[[[depriv*[Title/Abstract]] OR determinant*[Title/Abstract]] OR disparit*[Title/Abstract]] OR ineq*[Title/Abstract]] OR develop*[Title/Abstract]] OR health[Mesh] OR equit*[Title/Abstract] OR equalit*[Title/Abstract]]]] NOT [[[[[[[Americas[All fields]] OR India[All fields]] OR Asia, Southeastern[All fields]] OR Asia, Western[All fields]] OR Far East[All fields]] OR Australia[All fields]] OR Africa[All fields]]] NOT [Australian[All fields] OR Indi*[All fields] OR Uganda[All fields] OR Japan[All fields] OR Nigeri*[All fields]]]] NOT Chin*]] NOT USA] NOT United States of America] NOT Canada] NOT North America]] NOT Malawi]] NOT Keny*]] NOT Developing countries] NOT Bangladesh] NOT Philippines] NOT Pakistan]] NOT U.S] NOT America] NOT American
Search Strategy IBSS	[ti[policy] OR ti[intervention] OR ti[parenting] OR ti[community health planning] OR ti[charities] OR ti[daycare centres] OR ti[foster home care] OR ti[food assistance] OR ti[government funding] OR ti[tax exempt] OR ti[family supports] OR ti[child care centres] OR ab[policy] OR ab[intervention] OR ab[parenting] OR ab[community health planning] OR ab[charities] OR ab[daycare centres] OR ab[foster home care] OR ab[food assistance] OR ab[government funding] OR ab[tax exempt] OR ab[family supports] OR ab[child care centres]] AND [ti[child] OR ti[infant] OR ti[newborn] OR ti[early childhood] OR ti[prenatal] OR ab[child] OR ab[infant] OR ab[newborn] OR ab[early childhood] OR ab[prenatal]] AND [ti[depriv*] OR ti[determinant*] OR ti[disparit*] OR ti[ineq] OR ti[develop*] OR ti[health] OR ti[equit*] OR ti[equalit*] OR ab[depriv*] OR ab[determinant*] OR ab[disparit*] OR ab[ineq] OR ab[develop*] OR ab[health] OR ab[equit*] OR ab[equalit*]]

### Study selection

**Figure 1:**
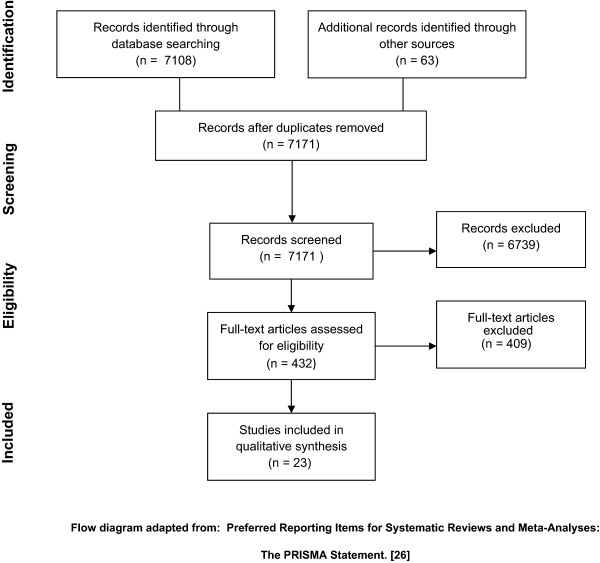
**Flow diagram of the number of records identified, included and excluded.** Flow diagram adapted from: Preferred Reporting Items for Systematic Reviews and Meta-Analyses: The PRISMA Statement [33].

Figure 1 shows the flow diagram mapping the number of records selected, included and excluded. The search carried out in PubMed retrieved 2361 articles, and the screening of the 2361 titles and abstracts was carried out by one reviewer (JM). After the first screening, 273 studies were selected and read by one reviewer (JM), leaving 6 studies which met the selection criteria. In this process the entire article was read revealing that the majority of the preselected studies described interventions which also included late childhood or adolescents or took place outside Europe and were therefore excluded. A second search was performed in IBSS which retrieved 4747 articles. The first screening of the titles and abstracts was carried out by one reviewer (JM). After the first screening, 159 studies were selected and read by one reviewer (JM), leaving 5 studies which met the selection criteria, for the same reason as described above. Four reports describing various early childhood interventions were retrieved from the grey literature. Two presented information on interventions which met our abovementioned selection criteria and two were excluded as there was not sufficient specific information on the evaluation. Finally twelve interventions published in the grey literature were selected.

### Data extraction, variables and analysis

The following information was extracted from each of the studies retrieved: the title and authors of the study and the year it was published, name of the intervention and country where it was delivered. Type of study design and objectives described, the domains of child development mentioned, target population and scope of the intervention, final sample size and age of the participants if these were children, were also included. Additional information was extracted on premises where it took place, staff which delivered the intervention and which activities were carried out, the evaluation performed, measured outcomes and results. This information was stored in a database as a complementary file. As the studies found were very diverse, these were summarised for comparison in Table 2 under the following headings: type of study design, intervention details and activities, target population, description of the sample including number of people in the intervention and control groups and age of the participants where pertinent. Information on the type of intervention-whether it was targeted or universal-was provided as well as its impact categorised as: a) developmental outcomes, b) parenting, c) health outcomes and d) outcomes of the intervention.

**Table 2:** **Outcomes of the interventions by evaluation design**

Design	Intervention details/Activities	Target	Evaluation sample description	Type	Impact	Outcomes
**RCT**	“The Positive Parenting Programme” [Triple P] developing parenting skills through: media, tip sheets, parent groups, self- directed and one to one activities, 5 intensity levels tailored to need	In Scotland, UK, evaluated among children 0-3	Various studies, sample sizes from 16 to 806	Proportionate universal	Parenting Health and development	Favourable outcomes in child behaviour. Abuse and injuries were reduced [19]
	“Preparing for Life” [PFL] improving school readiness from pregnancy until beginning of school by providing public health information, a support worker, materials and workshops. High treatment groups receive home visits from PFL trained mentors	Pregnant mothers and children living in disadvantaged communities in Ireland recruited between 2008-2010	High treatment: 115, low: 118. 0-7	Targeted Different treatment levels	Health	Limited improvement for maternal health behaviours. Favourable for parenting behaviours, higher immunisation rates, appropriate infant feeding patterns, better overall health. Children in the high treatment group showed higher level of fine motor skills and cognitive functioning [22]
	“Childhood Development Initiative-Early years” care and education programme delivered by specialised staff starting when children are aged 2–3 Parents participate in the Parents Plus Community Course and there is provision of quality childcare, home visits and activities for parents based on their specific needs are offered	All families living in an area of social disadvantage in Dublin, Republic of Ireland	When children were aged 2½-3, delivered in 2 waves, lasting 2 years	Targeted	Development	Fewer IG (Intervention Group) children had behavioural problems or high hyper-activity levels but not statically significant. They also scored significantly higher than the control group at mid and end phase on The Early Childhood Environmental Rating Scales. The Parents Plus Community Course was shown to improve the children’s home learning environment 2 years after the course was attended [22]
	“The Growing Child Parenting Programme” parent-directed child-centred monthly learning programme delivered through age-specific information and practical learning activities supported by tailored resources. Home delivered by trained visitors	Parents of children of children aged from birth to 5 years across Ireland, 2008-2009	IG: 216, CG: 208,	Proportionate universal	Development	The evaluation reported some positive effect on the domains of development but no statistically significant improvements at the present stage, however it showed greater parental efficacy [22]
	“Community Mothers Program” home visits by community mothers guided by a ‘family development nurse’ once a month. It focuses on health, nutrition and overall child development by emphasising on empowerment, parent capacity building and behavioural approaches illustrating alternatives in coping with child-rearing problems	First-time parents in deprived areas, Ireland and the UK [1983–present]	IG: 141, CG: 121. Age: 0-1	Targeted	Parenting Health	Significant more visits to the library, no significant difference in immunisation, dental checks, diet, breastfeeding or attendance to accident and emergency rooms. Intervention mothers were significantly more likely to check homework every night and more likely to disagree with the statement ‘children should be smacked for persistently bad behaviour’ [19]
	“The Social Support and Family Health Study”: postnatal support provided by seven home visits and additional telephone contacts	Women living in deprived districts who gave birth: 1/1/99-30/9/99, London, UK	Community group intervention: 184, health visitor int.:183, CG:364	Targeted	Parenting and health	There was no evidence of impact on child injury, maternal smoking or maternal depression of either intervention. There were different patterns of health service use and less anxious experiences of motherhood among IG women [36]
	Specialist home health visits received counselling on managing eating problems and parent–child interactions	Children with failure to thrive, most families depended on social welfare April 1994 -February 1996, UK	IG: 42, CG: 41. Age: 4–30 months	Targeted	Development Health care	Specialist health visitor intervention conferred no additional benefits but improved coordination in health care services use [37]
**Quasi-experiments with control group**	“Let’s Play in Tandem”, a compensatory education programme delivered by parents through play to foster one-on-one verbal interactions, a joint focus of attention, and scaffolding of the children’s learning providing children with prompts, demonstrations and encouragements	Socio-economically disadvantaged pre-schoolers from a “Sure Start” sample in Wales, UK	IG:30, CG:30, Mean age: 36.7 month	Targeted	Development	The intervention group outperformed matched controls in tests of academic knowledge, receptive vocabulary, inhibitory control and school readiness [19]
	“Incredible Years” weekly meetings during 12 weeks, 18–22 sessions training children on social skills, teacher training	Families with children at risk of conduct disorder, UK [2001-present]	Wales 153 families, Oxford: IG:44, CG:32	Targeted	Development	Intervention children exhibited fewer negative and submissive conducts and higher rates of positive-affect behaviours [19]
	“Sure Start” outreach, child care and home visits, support, healthcare advice, adding value to existing services	Low SES children, intensity varied in sites. UK, [2001–present]	IG: 5883, CG: 1879	Targeted	Parenting Development	Favourable outcomes in independence, social behaviour, reduced risk of negative parenting and a better home-learning environment. No improvements in language, immunisations or accidents [19]
	“Eager and Able to Learn” developmental movement experiences delivered in a group setting; a home learning package; workshops for parents and children; comprehensive training for the practitioners by Early Years specialists; 5 on-site support visits	Piloted in 14 settings, 2008–2009, Northern Ireland, UK	454 children 2–3 years old	Targeted	Development	Significant improvements in social and emotional development. Negative effect on emergent literacy skills. Positive parenting outcomes [22]
	Targeted work with parents by provision and fitting of safety equipment in addition to a population-wide education and information campaign provided across the whole locality	Families with children within disadvantaged Sure Start areas, UK	Children under five in the intervention ward. Assessment at two years	Targeted	Health	Over two years the proportion of children attending an A&E department reduced at a faster rate in the intervention wards [38]
	“First Parent Health Visitor Scheme” approximately 10 home visits by trained visitors beginning at third trimester until 8 months old	First time parents in deprived areas in the UK [1989–1998]	IG: 205, CG: 254	Targeted	Health	Significantly fewer accidents in the past 12 months [22]
	Intervention to prevent burn and scald injuries at home by individual-based information with an empowerment approach	Low SES mothers selected by health care services in Sweden	99 mothers of children under 7 months	Targeted	Health	The intervention had a significant impact on improving precautions, in relation to the comparison group [39]
**Mixed-methods**	Support and advice on breastfeeding by trained nurses on breastfeeding techniques	Mothers 5–12 days after delivery in Redbridge trust, UK	All mothers in the trust area	Targeted	Parenting Health	Mother’s perceptions were that they would not have continued without support and prevalence went from 60.5% to 67.45% [40]
	“Family Nurse Partnership” Using a psycho-educational approach it provides on-going, intensive support to young, first-time mothers and their babies trained nurses provide home visits from early pregnancy until child is 2	Low Socio Economic Status [SES] mothers, UK	Formative evaluation	Targeted	Health and development	Piloted at ten sites, evaluations was still underway when the report was published [19]
	Freephone parenting help line for parents: carers called back and offered additional services if the call taker felt that the parent may benefit	Parents who contacted a national parenting help line, 1999, UK	97 parents received support, 99 awaiting	Targeted	Parenting	Parents felt that their abilities had improved across the domains, particularly with regard to their ability to understand their children’s needs and their confidence in their parenting abilities. They scored more favourably on the General Health Questionnaire [41]
	“The Speech and Language Therapy” provided training and support developing an interagency organisational structure for inter professional collaboration between Early Years practitioners and speech and language therapists	Early Years [EY] staff and parents Dublin, Republic of Ireland	3 primary schools and 10 EY services: 77 parents, staff and others	Targeted	Development	12% of the boys and 28% of the girls were discharged with their speech and language within normal limits. Around half of the children required on-going therapy. Parents reported that their children were more ready for school as a result of the intervention and that their child was less likely to be bullied [22]
	“Ready Steady Grow” a programme to improve health and wellbeing to promote and support the parent–infant relationships. The centre based Parent–Child Psychological Support Programme component is delivered through 6 visits by the parent and baby over a 15-month period by specialist staff	Parents and children in designated deprived area, Dublin, Republic of Ireland	23 interviews and 58 surveys with stakeholders	Targeted	Parenting Development	Increased global and language development. No significant decrease in parenting stress and no effect on motor and personal-social development [22]
**Qualitative methods assesments**	Day care with highly qualified staff	Socially disadvantaged families at a EY centre, UK	IG: 11, CG: 10 mothers	Targeted	Parenting	Women who received a day care place at the centre were more likely to be in paid employment [42]
	The Developing Everyone’s Learning and Thinking Abilities [DELTA] parenting programme	Services for children in need and their families, Northern Ireland, UK	46 individual interviewees 32 parents participated in focus groups. 154 postal questionnaires	Targeted	Parenting Development	75% felt more confident as parents, 65% that it enhanced their child’s learning. 58% felt that it increased their knowledge on health issues [43]
	Limerick Lullaby project: music and singing providing an additional tool for communication	Women in a deprived area with an uncomplicated pregnancy, Republic of Ireland	6 women age 29-35	Targeted	Parenting	Mothers described it improved connection, communication, stress reduction, confidence building and foetal attachment [44]
	Baby “FAST” strengthening family relationships through a structured curriculum comprising arts and craft-based activities, small group discussion, a community meal and infant foot massage	Teenage mothers in a deprived area, London, UK	Seven teenage mothers and fathers	Targeted	Parent and	Qualitative research showed positive results in consolidating intergenerational bonds and trust [45]

## Results

### Description of intervention characteristics

We identified 23 interventions in total: 6 in the PubMed data base, 5 in IBSS and 12 in the grey literature. All but 1 intervention-delivered in Sweden-were provided in the United Kingdom and the Republic of Ireland (22). The studies identified were RCTs (7), quasi-experiments with control group (7) followed by mixed methods (5) and qualitative evaluations (4). These aimed to improve parenting abilities, however, some had additional components such as: day-care provision (3), improving housing conditions (2) and speech or psychological therapies (5). The majority of the interventions had an impact on domains of child development (14), on parent–child bonding (15) or on children’s health and injury prevention (11), however interventions could have an impact on more than one area. Two studies were universally proportionate interventions, and all others were aimed at children and families living in deprived areas.

### Intervention outcomes according to their study design

Table 2 shows the intervention studies identified categorised into four types of evaluation: RCTs, quasi-experiments with control group, studies with a mixed methods approach and qualitative methods assessments. The studies vary regarding their activities, targets, sample sizes and measured outcomes. Below, the programmes, their principal activities and outcomes are described according to their study design and type of outcome.

### Randomised controlled trials

#### Favourable parenting, health and developmental outcomes

Within the RCTs, the “Family Nurse Partnership” (FNP) [19], “The Positive Parenting Programme” (Triple P) [19] and “Preparing for Life” (PFL) [22] provided mothers living in deprived areas and their children with home visits delivered by trained nurses and visitors from early pregnancy until the child was 2, 3 and until school age, respectively. They offered intensive support using a psycho-educational approach and provided public health information, materials and workshops to develop parenting skills and child development. Triple P and PFL offer different levels of treatment and intensity. The programmes showed favourable outcomes for parenting behaviours, higher immunisation rates, appropriate infant feeding patterns and better overall health. In PFL, children in the high treatment group showed higher level of fine motor skills and cognitive functioning. Child injuries, abuse and neglect were also reduced. The “Childhood Development Initiative-Early Years” (CDI) care and education programme also provided specialised home visits and activities based on their specific needs. The CDI Initiative was targeted at parents with 2–3 year olds and provided quality day-care. Children in the Intervention Group (IG) scored higher on the Early Childhood Environmental Rating Scales, and the home learning environment was shown to improve [22].

#### Favourable for parenting outcomes

The “Growing Child Parenting Programme” a parent-directed programme supported by tailored resources and home visits, showed no statistically significant improvements at the present stage, however it showed greater parental efficacy [22]. The “Community Mothers Program” provided home visits by community volunteers once a month and focused on parent capacity building. Its evaluation showed there were significantly more visits to the library and parents did not agree with physical punishment [19].

#### Favourable for health service use outcomes

“The Social Support and Family Health Study” [36] and an intervention for children with failure to thrive [37] received postnatal support provided by specialist home visits and additional telephone contacts. Both programmes showed improved coordination and patterns in health care services use and “The Social Support and Family Health Study” which also provided further telephone assistance, showed that women in the IG had less anxious motherhood experiences.

### Quasi-experiments with control group

#### Favourable outcomes for cognitive development and school readiness

“Let’s Play in Tandem” [46] is delivered by parents through play to foster one-on-one verbal interactions, demonstrations and encouragements for three year olds. IG children outperformed the control group in academic knowledge, receptive vocabulary, inhibitory control and school readiness.

#### Favourable outcomes for socio-emotional development and parenting

“Incredible Years”, “Sure Start” [19], “Eager and Able to Learn” [22] provided children with training on social skills, support, workshops and advice for parents in addition to home visits. IG children who received the intervention exhibited fewer submissive behaviours. Parents showed a lower risk of negative parenting.

#### Favourable outcomes for reducing accidents and injuries

One programme provided in Sure Start areas in the UK offered targeted work involving parents in fitting safety equipment and provided an education and information campaign [38]. The “First Parent Health Visitor Scheme” and an intervention to prevent burns and scalding delivered in Sweden, also offered individual based information through trained visitors. Results showed there were significantly fewer accidents, visits to the Accident and Emergency (A&E) departments and interventions had an impact on improving safety conditions and precautions [19, 39].

### Mixed-methods

The types of activities and outcomes of the interventions evaluated by mixed methods varied: support offered by trained nurses and advice on breastfeeding [40] had positive outcomes as mothers stated they would not have continued without support. A phone parenting line [41] gave parents assistance and offered other additional services and they felt their parenting abilities had improved; they scored more favourably on the on the General Health Questionnaire than parents waiting for the service. “The Speech and Language Therapy” [22] provided training and support to Early Years practitioners, teachers and parents to promote speech and language development; approximately half of the children required on-going therapy, parents reported their children being more prepared for school.

### Qualitative research methods

#### Favourable parenting outcomes

Day-care with qualified staff was provided in an Early Years centre in a deprived area in London, UK. It was education-led, flexible in catering to families’ needs, and of a very high quality. Data collected through in-depth interviews suggested that the flexibility of day care provided was particularly important in allowing women to return to paid employment [42]. The Developing Everyone’s Learning and Thinking Abilities [DELTA] parenting programme [43], the Limerick Lullaby project [44], and the Baby “FAST” [45] pilot study all aiming to strengthen family relationships and mother and child bonding through music and arts and crafts, had positive outcomes in parenting abilities and their confidence as parents and fostering intergenerational bonds.

## Discussion

This review identified eleven intervention studies published in peer reviewed journals in Pubmed and IBSS databases and twelve identified in NICE's database. It showed that all but two interventions targeted children and families living in deprived areas. These were aimed at providing parents with emotional support and parenting skills or resources and materials enabling them as active agents in the interventions. They were delivered in families’ homes by specialised home visitors or multidisciplinary staff and in clinics by health care professionals or in community centres and churches through workshops and individual sessions. All selected interventions had undergone an evaluation.

The interventions with better outcomes combined various activities such as workshops and educational programmes for both parents and children beginning during early pregnancy and included home visits by specialised staff. These provided parents with training and material resources to enable them as active agents in intervention delivery; for example, PFL [22] or “Let’s Play in Tandem” [46] showed more positive results than “The Social Support and Family Health Study” [36] or the intervention aimed at children with failure to thrive which were based almost exclusively on home visits. However, “Sure Start” [19] and “Eager and Able to Learn” [22] with similar programmes and structures had mixed outcomes. It has been described in previous evaluation reports that “Sure Start” research teams faced methodological challenges and the number of Sure Start Local Programmes (SSLPs) increased substantially, reducing the opportunity to identify suitable comparison areas [47]. Programmes with better outcomes also included elements such as interagency participation. An example of these is “Incredible Years” a preventive intervention successful in reducing behavioural problems and negative parenting in highly disadvantaged community based settings delivered by regular Sure Start staff. Furthermore, interventions delivered by specialised professional home visitors, such as the CDI [22] had more impact on positive parenting and reducing negative and submissive conducts in children than programmes delivered by volunteers or other non-professional home visitors like the “Community Mothers Program” [19]. They also had better outcomes than programmes such as “The Speech and Language Therapy” (SLT) and “Ready Steady Grow” (RSG) [22], delivered after birth during shorter periods. RSG, aimed at children 3–18 months showed more favourable outcomes for speech and language development than SLT, delivered to children 2–6 years old. Other interventions with favourable outcomes in improving child behaviour and reducing abuse and neglect such as “Triple P” [19], for example, were tailored to meet the child and family’s needs and offered different levels and intensity of activities and support.

Targeted work with parents through provision and fitting of safety equipment providing information such as the “First Parent Health Visitor Scheme” [19], showed reduced accident rates and increased precautions, however these interventions were targeted at very specific outcomes and used less rigorous methods and comparison groups than the abovementioned interventions with wider scopes and sample sizes. The “Developing Everyone’s Learning and Thinking Abilities [DELTA] parenting programme” [43], “Limerick Lullaby Project” [44] and “Baby Fast” [45] focused on mother and child bonding had positive outcomes and were evaluated using qualitative research methods. Some RCTs and quasi-experiments with a control group had more primary and secondary outcomes with no significant differences but had larger sample sizes and in particular the first may have a stronger level of evidence [48]. As it has been described elsewhere, small imbalanced sample sizes may reduce the power to detect differences and makes the study vulnerable to chance variation [49].

Some of the interventions identified are also implemented in countries such as Australia, Canada and the United States of America (USA). The Family–Nurse Partnership has shown long-term beneficial effects in the USA. It was evaluated by three RCTs and showed higher reading and mathematics tests scores in IG children. Long term evaluations showed children had fewer sexual partners, less smoking and drinking or ingestion of dangerous substances. Injuries and abuse were also reduced as was criminality during later years. In the UK, the FNP, has recently undergone a formative evaluation: nurses’ and mother’s feedback was very positive and provided support for the argument that group FNP-delivered to mothers who were not eligible for FNP-has been received well over the whole time period of the programme and good links were being made with other services [50]. However, if further evaluations are carried out, the results may not be as positive as those in the USA because the health visitor system and a universally accessible primary care system are already in place in the UK [19]. The evaluation of “Sure Start” Australia discovered that that there were very little detectable difference between the Sure Start Local Programmes and Start-to-be communities on most of the dimensions measured by the evaluation [51], similarly to “Sure Start” in the UK.

“Incredible Years” UK which showed favourable outcomes for socio-emotional development and behaviour replicated the results [52] found by Webster-Stratton’s evaluation of “Head Start” in the USA: intervention children were observed to exhibit significantly fewer conduct problems, less noncompliance and more positive affect than control children. One year later, most of the improvements were maintained [53]. Therefore, interventions with similar components were able to obtain the same results in a different context. The long-term outcomes of these programmes are important as children who show early persistent signs of antisocial behaviour are at greater risk of later juvenile delinquency and social exclusion with higher societal costs [52].

The majority of interventions identified were targeted at children living in deprived areas; the interventions were aimed at reducing social inequalities in children’s health and development by improving outcomes across the different domains among the most deprived populations. Previous studies suggest that the living conditions for young families should allow mothers to start pregnancy in a health-promoting environment as inequalities in health and development become set relatively early in life. Parents, teachers, health policies and services provide key guidance leading to the development of healthy outcomes [54]. To achieve equity from the start, it is important to foster the acquisition of cognitive and non-cognitive skills, which are strongly associated with educational achievement and with a whole range of other outcomes including better employment, income and physical and mental health [21]. Delivering programmes and interventions in disadvantaged areas will possibly help reduce health inequalities in later life, adulthood and throughout the lifecourse. These may also help reduce the intergenerational transmission of health inequalities as social and economic inequities affecting previous generations present an important influence on children’s life-course, and affects their life chances and health. Growing up in relative poverty has a strong influence on health and other outcomes throughout life [29]. However it has also been argued that while targeted pre-school education programmes have been found to have long-lasting effects on the social trajectories of poor children, improving their educational levels and employment prospects, their life chances remain significantly poorer than those of advantaged children not in receipt of targeted support [55].

From a critical point of view, only two interventions offered a proportionate universal approach by targeting need within universal delivery. Nearly all interventions identified were targeted, offering selective provision of services to children showing early manifestations of a problem or were at-risk of developing a problem early in life, as defined by the Organisation for Economic Co-operation and Development [56]. These were aimed at reducing inequalities in health and development among people living in deprived areas but not at levelling the social gradient in health. Within the gradient, health is progressively better the higher the socioeconomic position of people and communities. Therefore, it is important to design policies that act across the whole gradient and to address the people at the bottom of the social gradient and the people who are most at risk as described in the Review of social determinants and the health divide in the WHO European Region [29]. In similar reviews the authors found that most of the parent/infant stimulation programs dealt with “high-risk” children or interventions which focused almost exclusively on downstream initiatives in deprived areas [57]. Furthermore, the studies which were not targeted did not describe in their findings whether they had a differential impact for disadvantaged groups. However, by focusing only on high-risk families’ health outcomes, interventions are less likely to reduce inequalities across the social gradient and may not provide the best conditions for all children in which to develop and reach their full potential [21, 58]. Effective programmes should be universally available, with particular efforts made to ensure that all populations are reached, including the traditionally hard to reach [59].

In this review, interventions were based on improving parenting skills. Much of the published literature on early years interventions focuses on providing parents with support to improve their child rearing skills. These promote parenting behaviours which improve child cognitive development and help improve child attachment as positive effects of well-developed interventions, as described elsewhere, persist beyond schooling and into adulthood. While these parenting interventions are important, it is also necessary to address the conditions of daily life which make positive parenting difficult [29]. This requires policies aimed at children through an explicit, multi-dimensional and integrated strategy [26] and investment in reducing child poverty, improved living conditions and quality of housing, for example [11].

High quality child care has been described as being crucial for children’s development [29, 60] and is seen as service provision in some countries: 85% of mothers with children in preschool were in paid employment in the early 1990s in Sweden, for example. In other countries it receives limited public funding, the quality and type of services being more diverse and access to high quality child care restrictive for families with lower incomes [31]. However, only three studies assessing the impact of child care were identified in this review. Previous reviews - based on intervention descriptions - found that children’s centres were increasing in number in the UK, as part of a strategy of social investment [61, 62]. Studies by Melhuish and colleagues found that high quality children’s centres appeared to reduce socioeconomic inequalities, as children from less advantaged backgrounds benefited more than those from more advantaged backgrounds. Preschool participation was associated with strong benefits for later educational and job outcomes [63, 64]. Similarly, Feinstein [6] found that RCT studies showed a clear benefit for disadvantaged children who attended high quality pre-school childcare provision. Effective pre-school provision in England and Northern Ireland has shown evidence of longer-term benefits for all children and as described in Currie [31] this evidence has influenced policy in countries such as Australia, Norway and the Republic of Korea. Furthermore, the “Abecedarian Project” and the “Perry Preschool Project” delivered in the USA which showed very positive results as described earlier had high-quality childcare and education components and were highly resourced [59].

### Limitations and strengths

The search strategy was designed to include as much relevant information as possible but there may be other documents describing interventions to reduce social inequalities in health which have not been collected in this review. The limited number of retrieved studies shows that although the number of publications in this field has increased over the years in Europe, there are still relatively few papers published in scientific journals. Furthermore, as only evaluated studies were selected this study is possibly only providing a partial picture of the interventions being delivered.

The results focused predominantly on countries in the UK and the Republic of Ireland compared to the Nordic countries, France, Germany and Italy and other parts of Europe which were not represented in the literature. No papers were excluded due to a language criterion. However, language may be a barrier to publication for non-English speaking countries and local interventions may not be of interest to international peer reviewed journals.

To our knowledge, this is the first systematic review attempting to identify early years interventions throughout Europe that are effective in addressing inequalities in health and development and their social determinants. The evidence collected may be useful for researchers or decision makers and programme managers involved in the design and development of interventions and their delivery. The procedures used ensured the validity of data extraction and would not have hindered capturing as many published articles as possible from different contexts in Europe in whatever language they were written. As we only included interventions that had been evaluated, we were able to assess their effectiveness. Only one of the selected interventions had a negative impact on one developmental domain and only two had non-significant outcomes for some of the activities. However, this is likely to reflect publication bias against publishing wholly non-significant findings.

## Conclusions

Interventions were heterogeneous in their study population and sample size targets, outcome measures and furthermore, there was a large divergence in the quality of their study design. Interventions with better outcomes and a higher level of evidence combined workshops and educational programmes for both parents and children beginning in early pregnancy and included home visits by specialised staff. More literature reviews focusing on the grey literature are needed to develop a larger evidence base on early childhood interventions. Further evaluation of early years interventions should be carried out especially in countries outside the UK within the European context. This review may be a useful tool to provide evidence on effective interventions for specific early childhood development and health outcomes.
